# Round Shoulders

**Published:** 1890-05

**Authors:** 


### ROUND SHOULDERS.

Round shoulders are almost unavoidably accompanied by weak lungs, but may be cured by the simple and easily performed exercise of raising one's self upon the toes, leisurely, in a perpendicular position, several times daily. Take a perfectly upright position, with the heels together, and the toes at an angle of forty-five degrees. Drop the arms lifelessly by the sides, animating and raising the chest to its full capacity muscularly, the chin well drawn in. Slowly rise up on the balls of the feet to the greatest possible height, thereby exercising all the muscles of the legs and the body ; come again into standing position without swaying the body backward out of the perfect line. Repeat this exercise first on one foot, then on the other.

